# CBX4 facilitates EV71 replication by SUMOylation and stabilizing 3D polymerase

**DOI:** 10.3389/fmicb.2026.1775950

**Published:** 2026-03-04

**Authors:** Rui Su, Yifan Niu, Aiping Sun, Tiesuo Zhao, Hui Wang

**Affiliations:** 1 Department of Immunology, School of Basic Medical Sciences, Henan Medical University, Xinxiang, China; 2 Xinxiang Engineering Technology Research Center of Immune Checkpoint Drug for Liver-Intestinal Tumors, Henan Medical University, Xinxiang, China; 3 Henan Collaborative Innovation Center of Molecular Diagnosis and Laboratory Medicine, School of Medical Technology, Henan Medical University, Xinxiang, China

**Keywords:** 3D polymerase, CBX4, Enterovirus 71, protein stabilization, SUMOylation

## Abstract

Enterovirus 71 (EV71) is a primary etiological agent of hand-foot-mouth disease (HFMD) in children under 5 years of age and can cause severe neurological disorders even death. Therefore, elucidating the infection mechanism and pathogenicity of EV71 is essential for developing more effective and targeted therapies to prevent and control EV71-associated diseases. Here, we initially reported that the SUMO E3 ligase CBX4 is important for EV71 replication. Furthermore, we found that CBX4 interacts with the EV71 3D polymerase, and overexpression of CBX4 significantly extends the half-life of 3D, whereas knockdown of CBX4 reduces the stability of 3D protein. Subsequent investigations demonstrated that CBX4 mediates both SUMOylation and ubiquitination modifications of 3D, and treatment with protein SUMOylation inhibitor 2-D08 remarkably depresses EV71 replication and the expression of ectopically transfected 3D. The regulatory role of CBX4 and the effect of 2-D08 were also observed in other enteroviruses, including coxsackievirus B3 (CVB3) and poliovirus 1 (PV1). These findings revealed that CBX4 facilitates EV71 infection through inducing SUMOylation and stabilization of 3D polymerase, hinting its potential as a novel target for antiviral development.

## 1 Introduction

Enterovirus 71 (EV71), a single positive-stranded RNA virus of Picornaviridae family, was first successfully isolated in 1969 from infants with nervous system disorders in California (Schmidt et al., 1974). Since then, the outbreaks of EV71 epidemic have been reported worldwide particularly in Asia-Pacific region, including Malaysia, Japan, Australia, Taiwan, and mainland China (Huang and Shih, 2014; Liu et al., 2015). EV71 infection causes hand-foot-and-mouth disease (HFMD), at the same time, the patients with severe HFMD develop into multiple neurological complications such as aseptic meningitis, brainstem encephalitis, polio-like paralysis and acute flaccid paralysis (Yi et al., 2017; Lee, 2016), posing an urgent public health threat. However, the precise mechanism of EV71 survival and replication are not entirely clear and no specific drugs are currently available.

The EV71 genome encodes both structural proteins VP1, VP2, VP3, and VP4, and non-structural regulatory proteins including 2A, 2B, 2C, 3A, 3B, 3C, and 3D. These viral components hijack cellular signal transduction pathway and weaken antiviral immune response to establish viral infection (Ang et al., 2021; Yuan et al., 2018). Among them, the 3D protein functions as an RNA-dependent RNA polymerase (RdRp). It synthesizes negative-strand RNA directly using viral genomic RNA as a template to generate double-stranded RNA (dsRNA), which is essential for the production of new viral genomes and proteins (Sun et al., 2012; Jiang et al., 2011). Recent researches have demonstrated that several host factors are involved in the regulation of EV71 infection through 3D. For example, SIRT1 deacetylates 3D, inhibiting its RNA polymerase activity and ultimately reducing EV71 replication (Han et al., 2016). Conversely, METTL3 interacts with 3D and promotes viral replication by enhancing its SUMOylation and ubiquitination modifications (Hao et al., 2019). Additionally, ANXA2 and the Ragulator-Rag complex facilitate EV71 replication by recruiting 3D along with host factor PI4KB to form viral replication organelles (Zhang et al., 2021; Wang et al., 2023).

CBX4 is a member of the Polycomb group (PcG) protein and a core component of Polycomb Repressive Complex 1 (PRC1) (Morey et al., 2012). It contains two key functional domains, one is N-terminal chromodomain, through which CBX4 binds to chromatin and exerts transcriptional repression via PRC1, and another is two SUMO-interacting motifs (SIMs), which confer its SUMO E3 ligase activity toward specific substrates (Ren et al., 2019; Liang et al., 2022). Through Polycomb-related or SUMOylation functions, CBX4 plays a critical role in occurrence and progression of multiple malignant tumors, including hepatocellular carcinoma, osteosarcoma, breast cancer, lung cancer and kidney cancer (Li et al., 2014; Wang et al., 2020; Sanyal et al., 2020; Zhao et al., 2024; Chen et al., 2023; Jiang et al., 2020). In addition, CBX4 utilizes its SUMO ligase activity to regulate epidermal homeostasis by modulating the expression of important factors such as EZH2, DNMT1 and BMI1, thereby inhibiting stem cell activation and differentiation (Luis et al., 2011; van Wijnen et al., 2021). In viral infection, CBX4 enhances the formation of phase-separated nuclear bodies and SUMOylation of EZH2, thus promoting latent infection of HIV-1, whereas inhibition or degradation of CBX4 induces the reactivation of HIV-1 latency (Wu et al., 2022; Chen et al., 2025). However, the effect of CBX4 in EV71 replication and pathogenesis remains unclear.

In this study, we identified CBX4 as a vital host protein in facilitating EV71 replication. Our results showed that CBX4 enhances the stability of viral 3D polymerase by mediating its SUMOylation and ubiquitination. Correspondingly, pharmacological inhibition of protein SUMOylation with 2-D08 reduced both 3D protein expression and EV71 replication. Moreover, 2-D08 effectively repressed the replication of coxsackievirus B3 (CVB3) and poliovirus 1 (PV1). Thus, our work reveals CBX4 as a novel host regulator and uncovers a critical mechanism of 3D polymerase SUMOylation in EV71 infection.

## 2 Materials and methods

### 2.1 Cell culture and transfection

Human embryonic kidney cell (HEK293T), human colon adenocarcinoma cell (HT29) and human cervical cancer cell (HeLa) were purchased from American Type Culture Collection (ATCC) (Manassas, VA, USA). Human rhabdomyosarcoma cell (RD) was obtained from China Center for Type Culture Collection (CTCC) (Wuhan, China). All the cells were cultured in DMEM medium with 10% fetal bovine serum, 1% penicillin–streptomycin and maintained at 37 °C in a 5% CO2 incubator.

For transfection, the cells were seeded into plate and transfected with indicated plasmids and Lipofectamine 2000 (Lipo2000) (Invitrogen, Carlsbad, CA, USA) mixture according to the manufacturer’s instructions.

### 2.2 Virus infection

The EV71 (Xiangyang-Hubei-09) (GenBank accession no. JN230523.1), CVB3 (Nancy strain) and PV1 (vaccine strain) were obtained as described previously (Su et al., 2020). The viruses were propagated in RD cells. For viral infection assays, the cells were infected with virus at indicated multiplicity of infection (MOI). After 2 h, the unabsorbed virus was washed off and the infected cells were cultured in no FBS DMEM medium for specific time.

### 2.3 Reagents

Rabbit anti-EV71 VP1 (PAB7631-D01P) antibody was purchased from Abnova (Taipei, Taiwan). Rabbit anti-CBX4 (A5532), anti-EV71 3D (A22651) and anti EV71-3C (A20687) antibodies were obtained from ABclonal Technology (Wuhan, China). Mouse anti-*β*-actin (66009-1-Ig), anti-GAPDH (60004-1-Ig) and anti-Myc (60003-2-Ig) antibodies and rabbit anti-HA (51064-2-AP) antibody were purchased from ProteinTech Group (Wuhan, China). Mouse anti-EV71 3D (GTX630193) antibody was obtained from GeneTex (Irvine, CA, USA). Mouse anti-Flag (F1804) and anti-HA (901514) antibodies were obtained from Sigma-Aldrich (St. Louis, MO, USA) and BioLegend (San Diego, CA, USA), separately. Inhibitors including UNC3866 (HY-100832), 2-D08 (HY-114166), cycloheximide (CHX) (HY-12320) and MG132 (HY-13259) were purchased from MedchemExpress (Monmouth Junction, NJ, USA).

### 2.4 Plasmids and constructions

The DNA fragments of EV71 2A, 2B, 2C, 3AB, 3C and 3D were amplified from the cDNA of EV71 and inserted into pCAGGS-HA or pEGFP-C1 vector. The CBX4, SUMO1, SUMO2, SUMO3, UB and Ubc9 genes were obtained from cDNA of HeLa cells and ligated with corresponding vectors.

### 2.5 Virus titer determination

The titers of EV71 in culture medium supernatants were determined by plaque assay. In brief, RD cells were plated in 24-well plate, and added with different diluent virus samples for 2 h. Then, the cells were washed with PBS and cultured in DMEM medium supplemented with 2% FBS and 1% agarose. Three days later, the cells were fixed with 4% paraformaldehyde for 30 min. After washing three times with PBS, the cells were stained with crystal violet dye. The viral titers (PFU/ml) in supernatants from each group were determined by averaging the plaque counts from three independent wells in a certain volume of diluent.

### 2.6 RNA extraction and quantitative PCR

Total RNAs were extracted from cultured cells with TRIzol reagent (Vazyme, Nanjing, China) and reverse transcription was performed with 1 μg RNA into cDNA using HiScript II Q Select RT SuperMix (Vazyme). Real-time quantitative PCR (qPCR) analysis was executed using ABI 7500 Fast real-time PCR system (Applied Biosystem, Carlsbad, CA, USA). The relative changes in gene expression were determined by 2^–ΔΔCt^ method. The primers used for qPCR were as follows: GAPDH, forward, 5’-AAGGCTGTGGGCAAGG-3′, reverse, 5’-TGGAGGAGTGGGTGTCG-3′; EV71 VP1, forward, 5’-GAGTTCCATAGGTGACAGC-3′, reverse, 5’-CTGTGCGAATTAAGGACAG-3′; CVB3, forward, 5’-CGGTACCTTTGTGCGCCTGTT-3′, reverse, 5’-GCGGTGCTCATCGACCTGA-3′; PV1, forward, 5’-CCGTATTGAGCCAGTATGTTTGT-3′, reverse, 5’-TAGCGAGTAGGTGGAGGTGTTCT-3′; HoxA10, forward, 5’-ACAAGAAATGTCAGCCAGAAAGG-3′, reverse, 5’-GATGAGCGAGTCGACCAAAAA-3′; HoxB9, forward, 5’-AGGCCGTGCTGTCTAATCAAA-3′, reverse, 5’-CGAGCGTGCAGCCAGTT-3′; HoxC13, forward, 5’-AAGGTGGTCAGCAAATCGAAAG-3′, reverse, 5′- TGGTACAAAGCGGAGACATAAATAGA-3′.

### 2.7 Western blotting and co-immunoprecipitation (co-IP)

The cells were lysed in RIPA buffer (50 mM Tris–HCl, 150 mM NaCl, 0.25% sodium deoxycholate, 1% NP-40, pH 7.4) and the protein extractions were centrifugated at 12,000 rpm for 10 min in a 4 °C centrifuge. After removing insoluble cell debris, 10% of lysate was collected as input sample, and the rest of supernatants were rotated with indicated antibodies overnight at 4 °C. Then, the samples were added with Protein G agarose (Santa Cruz, CA, USA) for 2 h to collect IP complex. For the SUMOylation assay, HEK293T cells were transfected with indicated plasmids for 24 h. After being lysed in RIPA buffer supplemented with 20 μΜ NEM (Sigma-Aldrich), the samples were immunoprecipitated with anti-GFP immunomagnetic beads (Yeasen, shanghai, China). The concentration of proteins was quantified by BCA Protein Assay Kit (Solarbio, Beijing, China). After loading and separating in 10% SDS-PAGE gel, the proteins were transferred to a NC membrane (Millipore, Billerica, MA, USA). After being blocked with 5% skim milk for 2 h at room temperature, the membrane was incubated with specific primary antibodies and secondary antibodies. The band was analyzed with a luminescent image analyzer (Amersham Imager 600, GE, MA, USA).

### 2.8 Cytoplasmic and nuclear proteins separation

The cytoplasmic and nuclear proteins were prepared with a Nucleoprotein Extraction Kit (Solarbio) according to the manufacturer’s instructions. Briefly, the collected cells were lysed with cytoplasmic protein extraction buffer and vortex for 15 s. After centrifugation at 12,000 g for 10 min in a 4 °C centrifuge, the prepared supernatants were collected for cytoplasm protein detection. Subsequently, the precipitations were treated with nuclear protein extraction buffer and vortex for 15 s. The samples were centrifugated at 12,000 g for 10 min in 4 °C and the lysates were collected for nuclear protein detection.

### 2.9 Cell viability measurement

The cell viability was measured by a cell counting kit 8 (CCK8) assay. RD cells with 2,000 cells per well were plated in 96-well plate and treated with inhibitor or virus. After 24 h, each well was added with 10 μL CCK8 solution and incubated for 1 h at 37 °C. The cell viability was detected at 450 nm absorbance.

### 2.10 Confocal microscopy

The cells were plated on 20-mm coverslips and treated with plasmids transfection or virus infection. After indicated time interval, the cells were fixed with 4% paraformaldehyde, permeabilized with 0.4% Triton X-100, and then blocked with 5% bovine serum albumin (BSA). After incubating with indicated primary antibodies overnight at 4 °C, the samples were stained with fluorescein-conjugated secondary antibodies for 1 h at room temperature. Nuclei were stained with DAPI (Roche, Basel, Switzerland). The samples were imaged with confocal laser scanning microscopy (Leica Microsystem, Wetzlar, Germany).

### 2.11 Lentivirus package and stable cell lines generation

The short-hairpin RNAs (shRNAs) target negative-control (NC) and CBX4 were inserted in pLKO.1 vector. The shRNA sequences were designed as follows, shNC, 5’-CAACAAGATGAAGAGCACCAA-3′; shCBX4, 5’-GAGTGGAGTATCTGGTGAAAT-3′. For lentivirus package, HEK293T cell were plated in 6 cm dishes and transfected together with 1.5 μg psPAX2, 0.5 μg pMD2.G and 2 μg pLKO.1 ligating with shNC or shCBX4 plasmids with Lipo2000. At 24 h and 48 h, the lentivirus supernatants from HEK293T cells were collected. After being infected with lentivirus for 48 h, RD, HEK293T and HT29 cells were selected by puromycin (2.5 μg/mL; Sigma-Aldrich). The knockdown efficiency of shRNA was evaluated by Western blotting.

### 2.12 Protein stability assay

HEK293T or RD cells were seeded in 6-well plate and subsequently transfected with indicated plasmids or infected with virus. After indicated time interval, the cells were added with CHX at the concentration of 100 μg/mL for different time points. The cell lysates were prepared for Western blot analysis. The relative protein level of 3D polymerase to internal control GAPDH was quantified by Image J software.

### 2.13 Statistical analysis

All experiments were reproducible and repeated at least three times. The data were presented as mean ± SD. Statistical analysis for comparison of two means was assessed by unpaired Student’s *t*-test. Statistical significance in protein stability assay was calculated by two-way ANOVA. Analyses were performed with Prism 8 software (GraphPad Software Inc., La Jolla, CA, USA). *p*-value < 0.05 was considered as statistically significant.

## 3 Results

### 3.1 Overexpression of CBX4 enhances the replication of EV71

**Figure 1:**
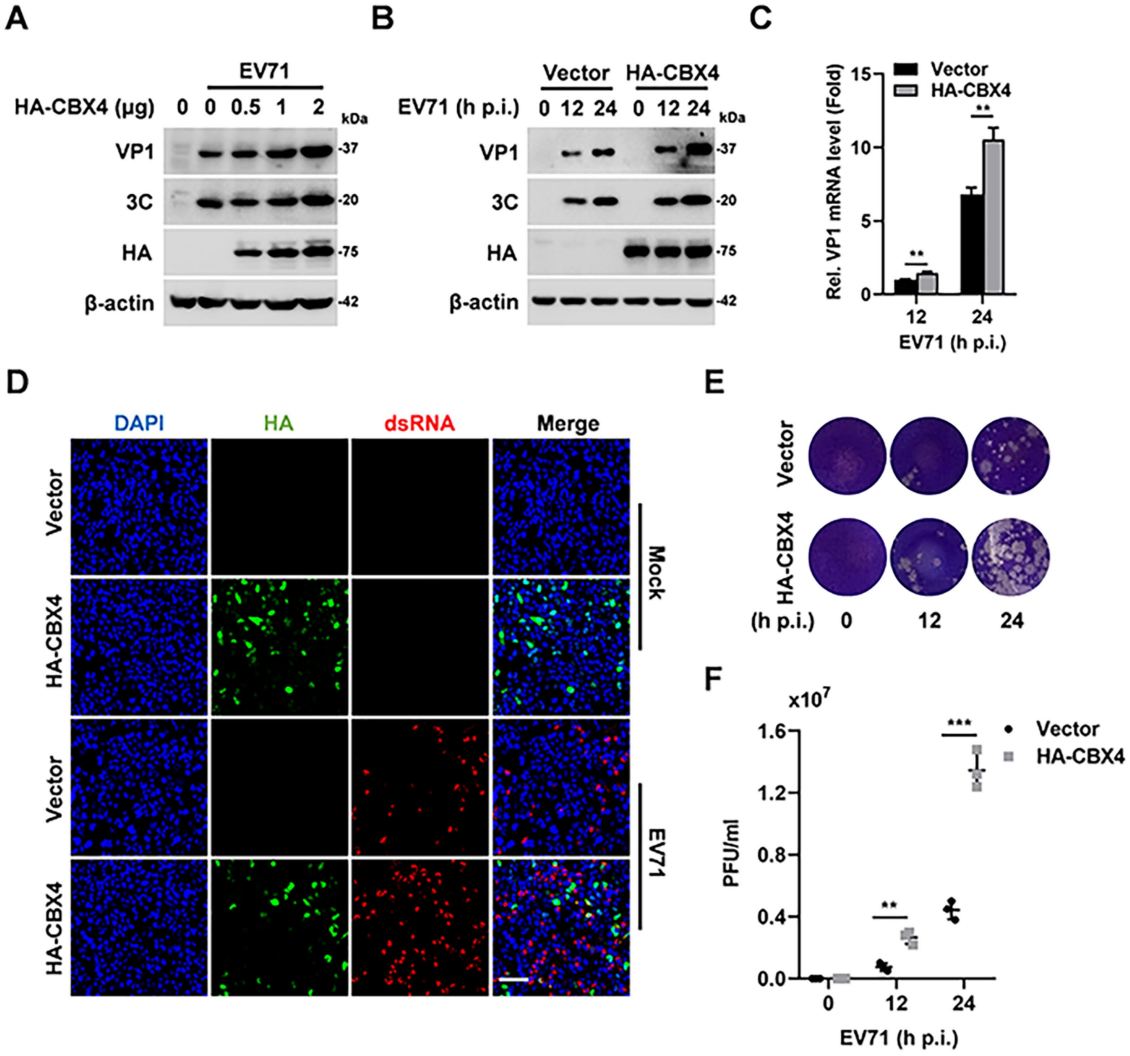
Overexpression of CBX4 promotes the replication of EV71. **(A)** RD cells were plated in 6-well plate and transfected with 0, 0.5, 1 or 2 μg HA-CBX4 expression plasmid for 24 h, after that, the cells were infected with EV71 (MOI = 1) for 24 h. The proteins were extracted from cell samples and detected by immunoblotting with VP1, 3C, HA and β-actin antibodies. **(B,C)** RD cells were transfected with vector or HA-CBX4 expression plasmid for 24 h, and then infected with EV71 (MOI = 1) for 0, 12, and 24 h. The protein levels of VP1, 3C, HA-CBX4 and β-actin were determined by Western blot analysis **(B)**. The level of VP1 mRNA was detected by qPCR, and the data were presented as fold induction relative to 12 h postinfection after transfection with vector **(C)**. **(D)** RD cells were plated in coverslips and transfected with vector or HA-CBX4 plasmid, 24 h later, the cells were infected with EV71 (MOI = 1) for 8 h. The staining of HA-CBX4 (green), dsRNA (red) and DAPI (blue) was analyzed by confocal microscopy. Bar = 50 μm. **(E,F)** RD cells were transfected with HA-CBX4 expression plasmid for 24 h, and then infected with EV71 (MOI = 1) for 12 and 24 h. The EV71 titers in supernatants were determined by plaque assay **(E)** and quantified **(F)**. The results are shown as mean ± SD. **p < 0.01, ***p < 0.001.

To interpret the function of SUMO E3 ligase CBX4 in EV71 replication and pathogenicity, we firstly assessed whether EV71 infection alters CBX4 expression. Following dose-dependent EV71 infection in RD cells, the levels of VP1 and 3C proteins were increased as expected, in contrast, CBX4 protein expression was maintained at a steady level (Supplementary Figure 1A). We then transfected increasing amounts of CBX4 plasmid in RD cells and found that with the elevation of CBX4 protein level, the replication of EV71 was markedly enhanced (Figure 1A). Furthermore, RD cells were transfected with CBX4 or control vector plasmid, and then infected with EV71 at various time points. Our results showed that the VP1 and 3C proteins and VP1 mRNA were induced upon EV71 infection, and this induction was upregulated by transfection of CBX4 compared with transfection of vector plasmid (Figures 1B,C). Immunofluorescence analyses indicated that overexpression of CBX4 promoted the expression of EV71 replication intermediate dsRNA distinctly (Figure 1D). At the same time, the effect of CBX4 on productive viral release was determined by plaque assay. We observed that CBX4 enhanced the viral titers in cell supernatants (Figures 1E,F). Overall, these results suggested that overexpression of CBX4 obviously facilitates the infection of EV71 in cells and release of infectious virus particles.

### 3.2 Knockdown of CBX4 reduces EV71 infection

**Figure 2:**
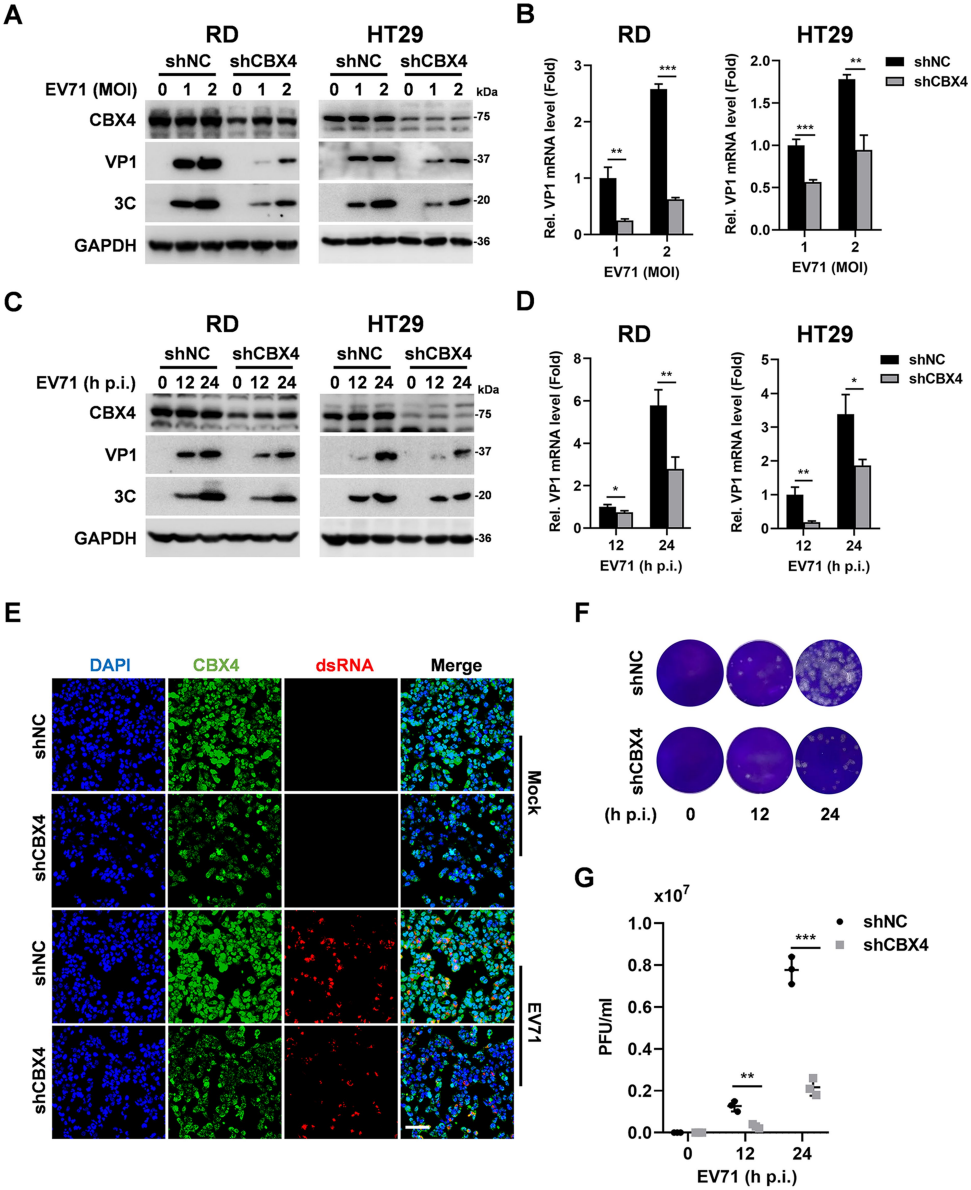
Knockdown of CBX4 reduces the replication level of EV71. **(A,B)** RD and HT29 cells stably expressing shNC or shCBX4 were generated and infected with EV71 at MOI of 0, 1, and 2 for 24 h. CBX4, VP1, 3C and GAPDH proteins expression were detected by Western blotting **(A)**. VP1 mRNA level was measured by qPCR, and the data were presented as fold induction relative to shNC cells infected with EV71 at MOI of 1 **(B)**. **(C,D)** RD and HT29 cells stably expressing shNC or shCBX4 were treated with EV71 (MOI = 1) for indicated time. Cell lysates were prepared, and the CBX4, VP1, 3C and GAPDH proteins were determined by immunoblotting **(C)**. The level of VP1 mRNA was measured by qPCR, and the results were presented as fold induction relative to 12 h postinfection in shNC cells **(D)**. **(E)** RD cells expressing shNC or shCBX4 were plated in coverslips infected with EV71 (MOI = 1) for 8 h. The staining of CBX4 (green), dsRNA (red) and DAPI (blue) was analyzed by confocal microscopy. Bar = 50 μm. **(F,G)** RD cells stably expressing shNC or shCBX4 were infected with EV71 (MOI = 1) for 12 and 24 h. The EV71 titers in supernatants were determined by plaque assay **(F)** and quantified **(G)**. The graphs are shown as mean ± SD. *p < 0.05, **p < 0.01, ***p < 0.001.

Moreover, we constructed lentiviruses expressing shNC or shCBX4 to transduce RD cells. As expected, shNC transduction did not significantly affect EV71 replication compared to non-transduced cells (Supplementary Figure 1B), and shCBX4 effectively attenuated CBX4 expression (Supplementary Figure 1C). Additionally, HT29 stably expressing shNC or shCBX4 cell lines were also generated. After reducing CBX4 expression, the cytopathic effect caused by EV71 was relieved in comparison to shNC expressing cells (Supplementary Figure 1D). The infection of RD and HT29 stable shNC cells with different doses of EV71 led to a robust increase in the VP1 and 3C proteins and VP1 mRNA, but these productions were depressed in shCBX4-treated cells (Figures 2A,B). Furthermore, RD and HT29 cells were infected with EV71 for different time points. Compared with shNC in RD and HT29 cells, the expression of VP1 and 3C proteins and VP1 mRNA were reduced in the presence of shCBX4 across the time course (Figures 2C,D). These results indicated that knockdown of CBX4 inhibits EV71 replication in a does- and time-dependent manner. Immunofluorescence analyses illustrated that knockdown of CBX4 diminished the expression of EV71 dsRNA (Figure 2E). Plaque assay revealed that knockdown of CBX4 restrained viral release in the culture medium supernatants (Figures 2F,G).

CBX4 is critical for EV71 infection, which raises the question of whether other enteroviruses also utilize CBX4 to regulate their replication. We showed that similar with EV71, the replication levels of CVB3 and PV1 decreased upon knockdown of CBX4 (Supplementary Figures 1E,F), suggesting that CBX4 also supports the replication of other enteroviruses. Taken together, the data revealed that CBX4 not only plays a vital role in EV71 replication, but also extends to the other enteroviruses.

### 3.3 CBX4 regulates EV71 replication through SUMOylation E3 ligase function

**Figure 3:**
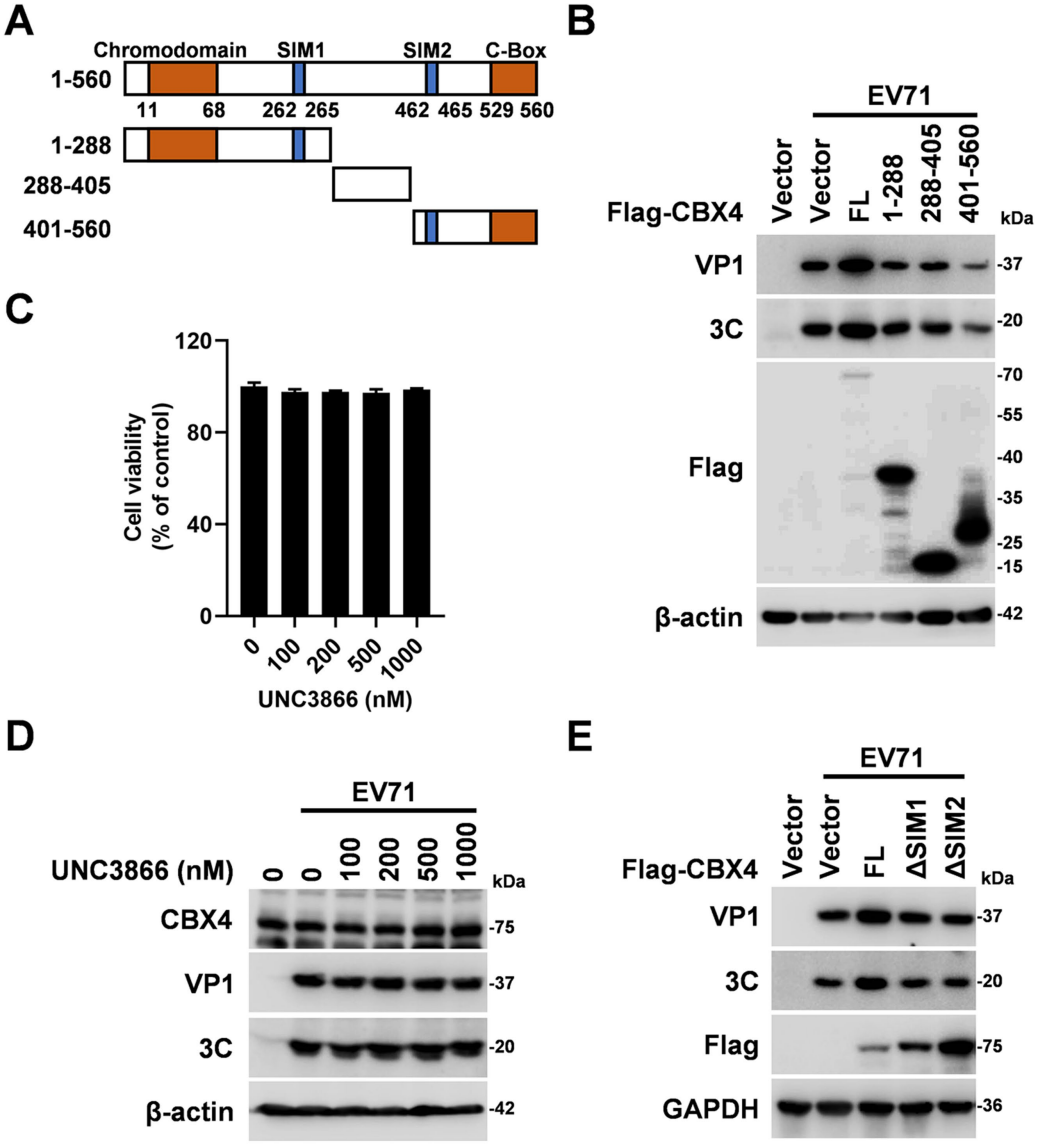
Effect of CBX4 and its mutans on EV71 replication. **(A)** The schematic diagram of CBX4 truncations construction. **(B)** RD cells were transfected with Flag-CBX4 FL, Flag-CBX4 1-288, Flag-CBX4 288-405 or Flag-CBX4 401-560 plasmids for 24 h, and then infected with EV71 (MOI = 1) for 24 h. VP1, 3C, β-actin and Flag-tagged proteins were determined by immunoblotting. **(C)** RD cells were treated with UNC3866 for 24 h at different concentrations (0, 100, 200, 500, and 1,000 nM), the cell viability was detected by CCK8 assay, the results were shown as percentage relative to control. **(D)** RD cells were added with UNC3866 (0, 100, 200, 500, and 1,000 nM) and EV71 (MOI = 1) for 24 h. The levels of CBX4, VP1, 3C and β-actin were determined by Western blot analysis. **(E)** RD cells were transfected with Flag-CBX4, Flag-CBX4 ΔSIM1 or Flag-CBX4 ΔSIM2 plasmids for 24 h, and then infected with EV71 (MOI = 1) for 24 h. The expressions of VP1, 3C, β-actin and Flag-tagged proteins were determined by immunoblotting with targeted antibodies. FL: full length.

To delineate the specific domain of CBX4 involved in EV71 replication, Flag-tagged CBX4 full length, 1-288 aa, 288-405 aa, and 401-560 aa truncation plasmids were constructed (Figure 3A). RD cells were subsequently transfected with these constructs for functional analysis. In contrast to CBX4 full length, which promoted EV71 VP1 and 3C proteins expression, 1-288, 288-405, and 401-560 mutants had no significant effect (Figure 3B), revealing that only full length of CBX4 is associated with the EV71 replication. We then investigated whether the impact of CBX4 on EV71 replication depended on its Polycomb-related or SUMO E3 ligase function. RD cells were treated with an CBX4 chromodomain-histone interaction antagonist (Stuckey et al., 2016), UNC3866 at different concentrations. Cell viability assays showed that the activity of RD cells remained stable throughout these concentration ranges (Figure 3C). Importantly, UNC3866 treatment upregulated known PRC1 target genes such as HoxA10, HoxB9 and HoxC13 (Supplementary Figure 2), providing functional evidence that it effectively inhibits the chromodomain of CBX4. We observed that UNC3866 did not impair the replication of EV71 (Figure 3D). Compared with full-length CBX4, its mutants defective in SUMO ligase activity (lacking SIM1 or SIM2) did not induce the upregulation of VP1 and 3C when overexpressed (Figure 3E). Therefore, the SUMOylation E3 ligase rather than Polycomb-related function of CBX4 is essential for the replication of EV71.

### 3.4 CBX4 interacts with EV71 3D polymerase

**Figure 4:**
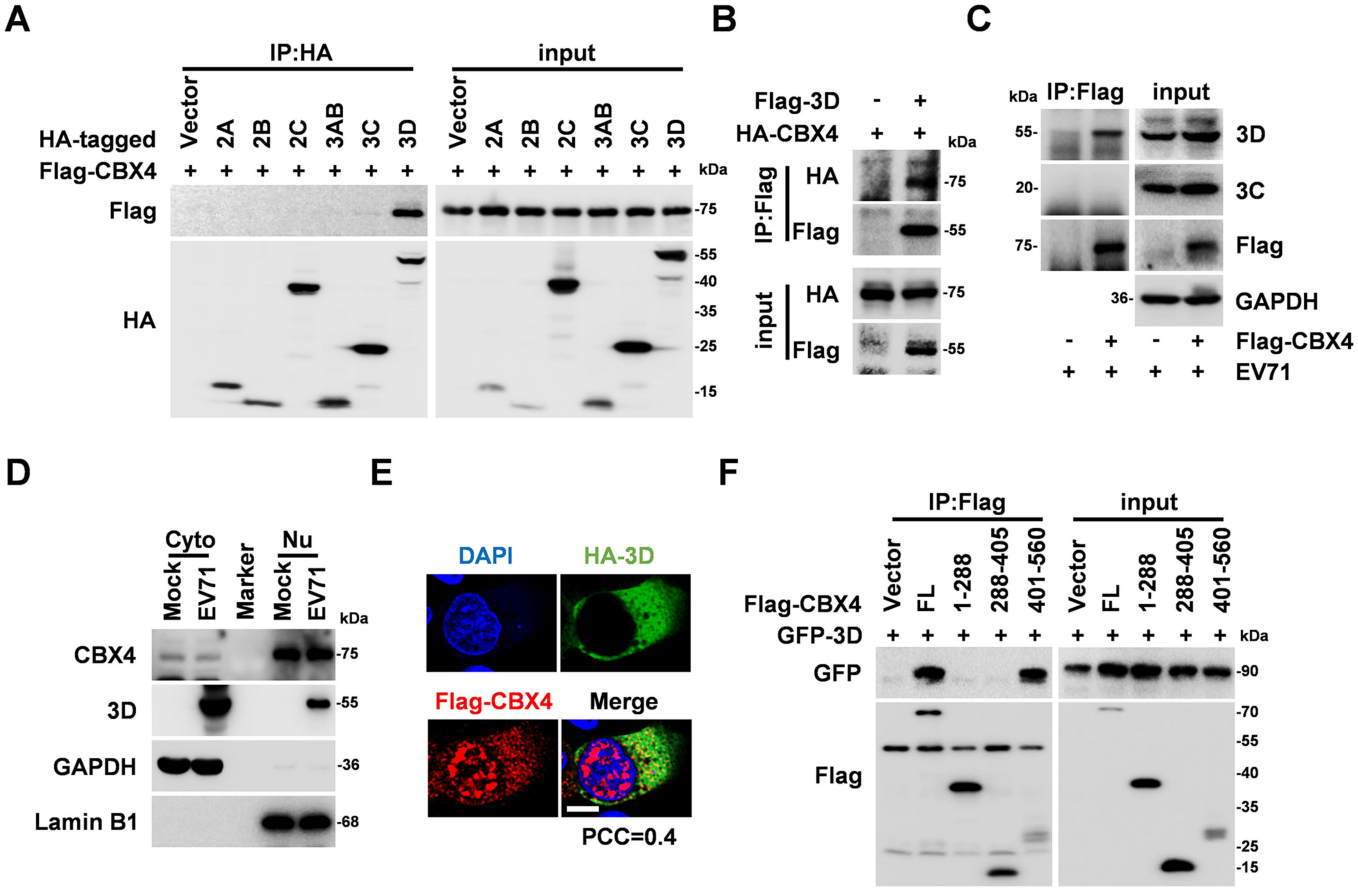
CBX4 interacts with EV71 3D polymerase. **(A)** HEK293T cells were transfected with the plasmids of HA-2A, HA-2B, HA-2C, HA-3AB, HA-3C, or HA-3D with Flag-CBX4 as indicated. Whole-cell lysates were prepared and immunoprecipitated with HA antibody. The expression of HA-tagged proteins and Flag-CBX4 in IP complex and input samples were determined by immunoblotting with HA and Flag antibodies. **(B)** HEK293T cells were transfected with Flag-3D and HA-CBX4 plasmids, and immunoprecipitated with Flag antibody. The IP complex and input samples were analyzed by Western blotting with antibodies target to HA and Flag. **(C)** RD cells were plate in 10 cm dish and transfected with vector or Flag-CBX4 expression plasmid, 24 h later, the cells were infected with EV71 (MOI = 1) for 12 h. The cell lysates were prepared and immunoprecipitated with Flag antibody. The protein levels of 3D, 3C, Flag-CBX4 in IP complex and 3D, 3C, Flag-CBX4, GAPDH in input samples were measured by immunoblotting. **(D)** RD cells were treated with mock or EV71 (MOI = 1) for 12 h. The cells were collected and lysed for preparing cytoplasmic proteins (Cyto) and nuclear proteins (Nu). The expressions of CBX4, 3D, GAPDH, and Lamin B1 were analyzed by Western blotting. **(E)** HeLa cells were transfected with HA-3D and Flag-CBX4 plasmids. The cells were stained with HA (green), Flag (red) antibodies and DAPI (blue) for confocal microscopy imaging. The Pearson’s colocalization coefficient (PCC) was determined using image J software. Bar = 20 μm. **(F)** HEK293T cells were co-transfected with GFP-3D and Flag-CBX4 FL, Flag-CBX4 1-288, Flag-CBX4 288-405 or Flag-CBX4 401-560 plasmids as indicated. The cells were collected and immunoprecipitated with Flag antibody, the expression of GFP-3D and Flag-tagged proteins in IP complex and input samples were measured with Flag and GFP antibodies.

The possible mechanism by which CBX4 regulates EV71 replication was evaluated by a co-IP screening experiment among several EV71 non-structural proteins and CBX4. HEK293T cells were co-transfected with the plasmids expressing Flag-tagged CBX4 and HA-tagged EV71 2A, 2B, 2C, 3AB, 3C, and 3D, respectively. We only detected the expression of Flag-CBX4 in HA-3D-IP complex (Figure 4A), demonstrating that 3D specifically interacts with CBX4. Additionally, the combination of CBX4 and 3D was also proved by co-transfected with Flag-3D and HA-CBX4 plasmids (Figure 4B). Furthermore, RD cells were transfected with Flag-CBX4 expressing plasmid, and then infected with EV71. It was shown that CBX4 was association with 3D rather than 3C generated by EV71 replication (Figure 4C). Previous studies demonstrated that CBX4 mainly takes effect in nuclear compartment (Zhao et al., 2024; Gu et al., 2024), however, immunofluorescence analysis of another study indicated that CBX4 is also expressed in cytoplasm (Liang et al., 2022). As a single positive-stranded RNA virus, the life cycle of EV71 is completed in cytoplasm (van der Linden et al., 2015). Therefore, we performed a nucleus and cytoplasm isolation experiment of RD cells after non-infection and infection with EV71. Our results showed that CBX4 was predominantly distributed in the nucleus, while a small apart was localized in the cytoplasm regardless of EV71 infection (Figure 4D), which provides evidence for CBX4 to regulate EV71 infection in cytoplasm. Next, we found the co-localization of 3D and CBX4 in the cytoplasm by immunofluorescence analysis (Figure 4E). Moreover, the interaction region of 3D and CBX4 was explored, and co-IP results revealed that 3D combined with the 401-560 aa of CBX4 (Figure 4F). Altogether, these findings consistently demonstrated the interaction between CBX4 and EV71 3D protein.

### 3.5 CBX4 enhances the stability of EV71 3D polymerase

**Figure 5:**
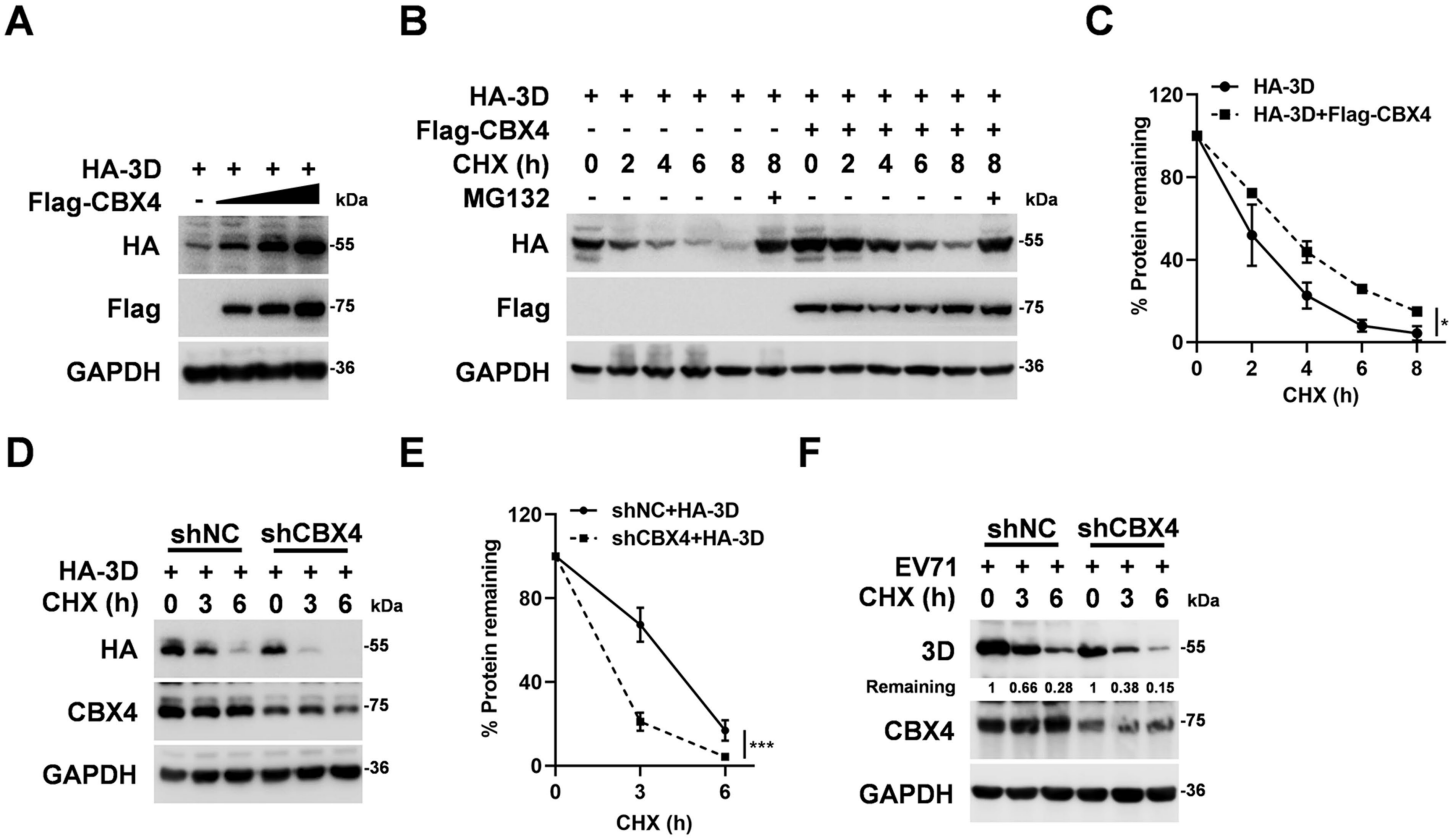
CBX4 enhances the stability of 3D protein. **(A)** HEK293T cells were co-transfected with 1 μg HA-3D and different doses of Flag-CBX4 plasmids as indicated. Cell lysates were prepared after 24 h transfection, and the levels of HA-3D, Flag-CBX4 and GAPDH were detected by Western blot analysis. **(B,C)** HEK293T cells were transfected with HA-3D alone or together with Flag-CBX4 plasmid for 24 h. The cells were treated with CHX for 0, 2, 4, 6, 8 h or MG132 and CHX combination for 8 h. HA-3D, Flag-CBX4 and GAPDH were determined by Western blotting **(B)**, and the HA-3D protein levels relative to GAPDH were quantified with Image J software **(C)**. **(D,E)** HEK293T stable expression shNC or shCBX4 cells were transfected with HA-3D plasmid for 24 h and then treated with CHX for 0, 3, and 6 h. HA-3D, CBX4 and GAPDH were detected by Western blotting **(D)**, and HA-3D protein levels relative to GAPDH were quantified with Image J software **(E)**. **(F)** RD cells stably expressing shNC or shCBX4 were infected with EV71 for 24 h and then added with CHX for 0, 3, and 6 h. The expression of 3D, CBX4 and GAPDH were detected by immunoblotting, and 3D protein levels relative to GAPDH were quantified with Image J software. The results are shown as mean ± SD. *p < 0.05, ***p < 0.001.

We transfected a gradient increased Flag-CBX4 plasmid together with a constant amount of HA-3D plasmid in HEK293T cells and found that the expression of 3D was markedly upregulated accompanied by the increase of CBX4 expression (Figure 5A), which hinted the possibility of CBX4 in stabilizing 3D protein. Subsequently, CHX chase experiment was carried out to measure the function of CBX4 in 3D stability. HEK293T cells were transfected with HA-3D plasmid alone or co-transfected HA-3D and Flag-CBX4 plasmids jointly, and then treated with the protein synthesis inhibitor CHX at different time points, as well as proteasome inhibitor MG132 and CHX together. The results indicated that co-transfection of CBX4 and 3D prolonged the half-life of 3D, and MG132 treatment attenuated 3D degradation (Figures 5B,C). We next investigated the effect of CBX4 knockdown on the influence of 3D half-life. CHX chase assay demonstrated that knockdown of CBX4 reduced the stability of 3D (Figures 5D,E). Moreover, RD stable shNC or shCBX4 cells were infected with EV71 and treated with CHX, the results showed that knockdown of CBX4 correspondingly diminished the stabilization of the 3D protein induced by EV71 infection (Figure 5F). Collectively, these data disclosed that CBX4 augments the half-life of EV71 3D polymerase.

### 3.6 CBX4 regulates the SUMOylation and ubiquitination of 3D

**Figure 6:**
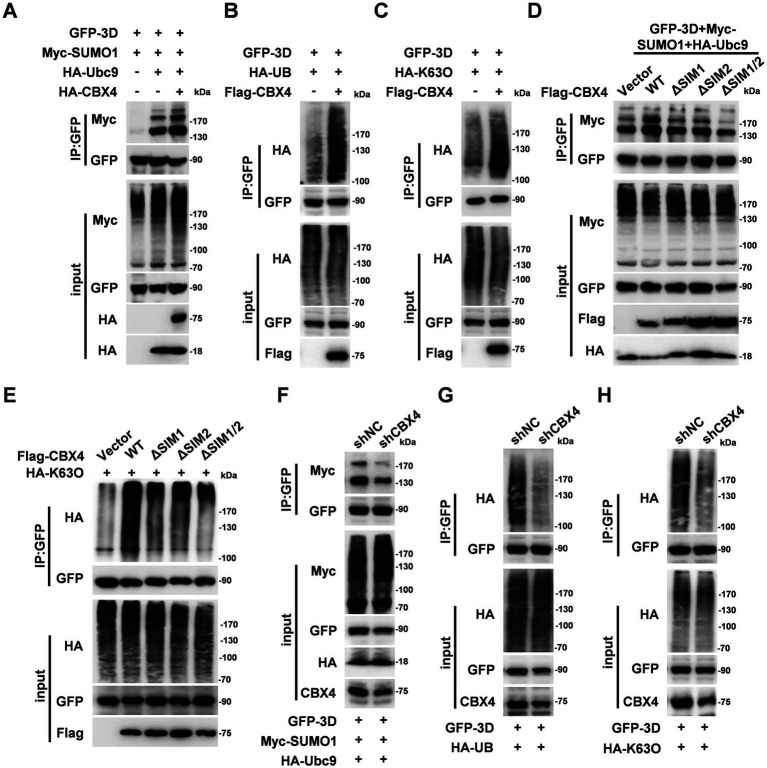
CBX4 enhances the SUMOylation and ubiquitination of 3D. **(A)** HEK293T cells were transfected with GFP-3D, Myc-SUMO1, HA-Ubc9 and HA-CBX4 plasmids as indicated, and immunoprecipitated with anti-GFP immunomagnetic beads. The IP complex was analyzed by antibodies target to Myc and GFP, and the expression of Myc-SUMO1, GFP-3D, HA-Ubc9 and HA-CBX4 proteins in input samples were measured with Myc, GFP and HA antibodies. **(B,C)** HEK293T cells were transfected with GFP-3D, HA-UB/K63O and Flag-CBX4 plasmids for 24 h, the cell lysates were immunoprecipitated with anti-GFP immunomagnetic beads. The IP and input complex were analyzed with indicated antibodies. **(D)** HEK293T cells were transfected with GFP-3D, Myc-SUMO1, HA-Ubc9 and Flag-CBX4 WT or different mutant plasmids, and immunoprecipitated with anti-GFP immunomagnetic beads. The IP and input samples were immunoblotted with indicated antibodies. **(E)** HEK293T cells were transfected with GFP-3D, HA-K63O and Flag-CBX4 WT or different mutant plasmids, and immunoprecipitated with anti-GFP immunomagnetic beads. The IP and input samples were immunoblotted with indicated antibodies. **(F)** HEK293T stable expression shNC or shCBX4 cells were transfected GFP-3D, Myc-SUMO1 and HA-Ubc9 plasmids. Twenty-four hour later, the cell lysates were prepared and immunoprecipitated with anti-GFP immunomagnetic beads. The expression of Myc-SUMO1 and GFP-3D in IP complex and Myc-SUMO1, GFP-3D, HA-Ubc9 and CBX4 in input samples were determined by immunoblotting. **(G,H)** HEK293T stable expression shNC or shCBX4 cells were transfected GFP-3D, HA-UB/HA-K63O plasmids for 24 h, the cell lysates were immunoprecipitated with anti-GFP immunomagnetic beads. The IP and input complex were analyzed with indicated antibodies.

It is previously reported that SUMO1-mediated SUMOylation and K63-linked ubiquitination upregulates the stabilization of 3D (Liu et al., 2016). Since CBX4 is a SUMO E3 ligase, and SUMOylation interacts with ubiquitination, the role of CBX4 on 3D SUMOylation and ubiquitination was examined. Firstly, we detected the SUMO1-based SUMOylation of 3D by overexpression of Ubc9, and such 3D-linked SUMOylation was notably enhanced upon co-transfection of Ubc9 and CBX4 (Figure 6A). Moreover, 3D-linked SUMO2 and SUMO3 levels were also upregulated by CBX4 (Supplementary Figures 3A,B). The ubiquitination levels of 3D were investigated. We found that overexpression of CBX4 enhanced whole ubiquitination and K63-linked ubiquitination of 3D (Figures 6B,C). Next, we determined the role of CBX4 SIM motifs in regulation 3D SUMOylation and ubiquitination. After the loss of either SIM1 or SIM2, the SUMOylation and k63-linked ubiquitination levels of 3D decreased, and when both SIM1 and SIM2 were absent, the SUMOylation and k63-linked ubiquitination levels of 3D were further reduced (Figures 6D,E). In addition, HEK293T stable shNC or shCBX4 cells were transfection with 3D, SUMO1 and Ubc9 expressing plasmids. The results showed that knockdown of CBX4 inhibited the SUMOylation level of 3D (Figure 6F). Meanwhile, we also detected that knockdown CBX4 significantly reduced the whole ubiquitination and K63-linked ubiquitination levels of 3D (Figures 6G,H). These data together suggested that CBX4 is involved in the regulation 3D SUMOylation and ubiquitination.

### 3.7 SUMOylation inhibitor 2-D08 suppresses 3D expression and EV71 replication

**Figure 7:**
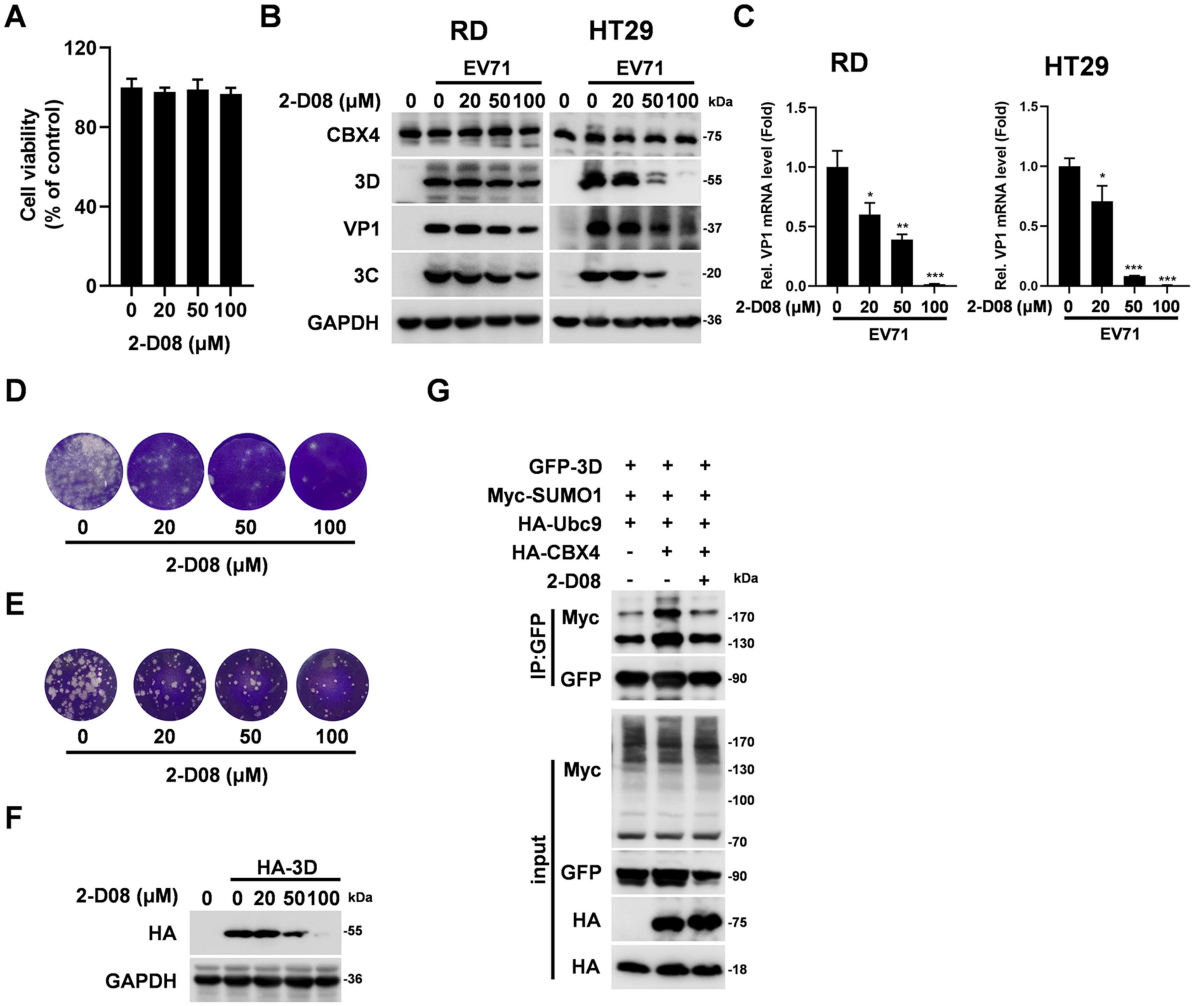
SUMOylation inhibitor 2-D08 suppresses EV71 replication. **(A)** RD cells were added with 0, 20, 50, and 100 μΜ 2-D08 for 24 h, the cell viability was determined by CCK8 assay. The results were shown as percentage relative to control. **(B–E)** RD or HT29 cells were treated with EV71 (MOI = 1) and 2-D08 (0, 20, 50, 100 μM), after 16 h, the supernatants and cells were collected. The expression levels of CBX4, 3D, VP1, 3C and GAPDH proteins were detected by Western blotting **(B)**, the mRNA level of VP1 was determined by qPCR assay **(C)**, the viral titers in supernatants from RD **(D)** or HT29 **(E)** cells were measured by plaque assay. **(F)** HEK293T cells were transfected with HA-3D plasmid for 6 h, and then treated with different concentrations of 2-D08 (0, 20, 50, 100 μM) for 24 h. The protein levels of HA-3D and GAPDH were detected by immunoblotting. **(G)** HEK293T cells were transfected with GFP-3D, Myc-SUMO1, HA-Ubc9 and HA-CBX4 plasmids for 6 h, and then treated with 2-D08 (50 μM) for 24 h. The cell lysates were immunoprecipitated with anti-GFP immunomagnetic beads. The IP and input complex were analyzed with indicated antibodies. The graphs are shown as mean ± SD. *p < 0.05, **p < 0.01, ***p < 0.001.

**Figure 8:**
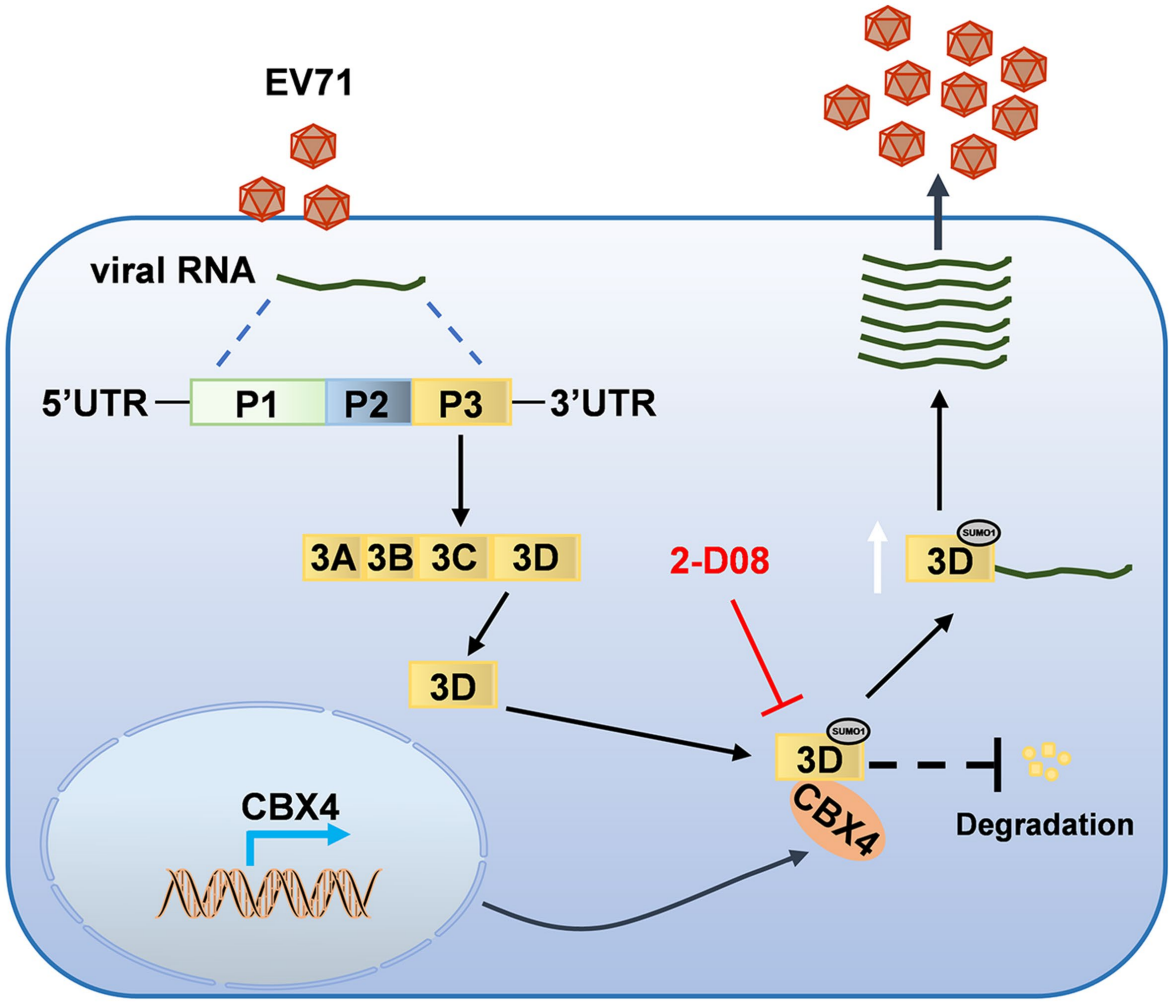
A model illustrating how CBX4 promotes EV71 replication. Host SUMO E3 ligase CBX4 interacts with EV71-encoded 3D polymerase and induces the SUMOylation of 3D, which results in the increased stability of 3D protein and EV71 replication. Pharmacological inhibition of protein SUMOylation with 2-D08 diminishes the SUMOylation and expression of 3D, and ultimately affects the replication of EV71.

Based on the above observations, we want to determine the regulatory relationship between SUMOylation and EV71 replication. We selected a SUMOylation inhibitor 2-D08, which is able to prevent SUMO transfer from Ubc9-SUMO thiol to substrates and inhibit the SUMOylation of substrate proteins (Kim et al., 2014). Firstly, the influence of different concentrations of 2-D08 on cell viability was evaluated by CCK8 assay. The results indicated the cells were stable in these concentrations of 2-D08 (Figure 7A). The function of 2-D08 on the replication of EV71 was dissected in RD and HT29 cells. With the increase of 2-D08 treatment concentration, the cell viability diminished by EV71 were significantly enhanced (Supplementary Figure 4A), the 3D, VP1, and 3C protein levels were decreased progressively (Figure 7B), as well as the VP1 mRNA was gradually declined (Figure 7C). The virus copy number (Supplementary Figure 4B) and viral titers (Figures 7D,E) in cell supernatants were also repressed by 2-D08. Besides, 2-D08 effectively depressed the viral RNA expression of CVB3 and PV1 (Supplementary Figures 4C,D) and restored the cell viability reduced by CVB3 and PV1 (Supplementary Figures 4E,F). We next explored the effect of 2-D08 on ectopically transfected 3D expression. HEK293T were added with different concentrations of 2-D08 and transfected with HA-3D expressing plasmid, the expression level of 3D was reduced by 2-D08 (Figure 7F). In addition, 2-D08 reduced the CBX4-mediated SUMOylation of 3D (Figure 7G). Thus, inhibition of SUMOylation of cell declines 3D protein level and EV71 replication capacity. In general, these findings indicated a mechanism by which CBX4 interacts with EV71 3D polymerase to mediate SUMOylation of 3D and enhance the stability of 3D, leading to facilitating the replication of EV71 in the end (Figure 8).

## 4 Discussion

The viruses usually hijack certain cellular pathway to interfere cellular physiological process and antiviral immune response, thereby creating a suitable microenvironment that is conductive to their replication and survival (Swain et al., 2022; Jin et al., 2018). Thus, understanding the interaction between viruses and host proteins will help us to identify potential drug targets and develop novel antiviral strategies. Here, we described the involvement of CBX4 in facilitating EV71 replication. Our research disclosed the mechanism of CBX4 in enhancing SUMOylation and stability of EV71 3D. Notably, diminishing the cellular SUMOylation system by 2-D08 resulted in the attenuation of 3D expression and EV71 replication. These investigations demonstrated the importance of CBX4 in EV71 replication and reveal a novel mechanism of how viruses utilize host protein to achieve infection.

It has been reported that PRC1 complex or PRC1 components are associated with viruses infection, such as PRC1 promotes Human cytomegalovirus (HCMV) lytic DNA replication in a enzymatically independent way (Svrlanska et al., 2019), RYBP facilitates the establishment of Kaposi’s sarcoma-associated herpesvirus (KSHV) latency following *de novo* infection (Lee and Toth, 2022), and CBX4 contributes to HIV-1 latency by forming phase-separated nuclear bodies and mediating SUMOylation of EZH2 (Wu et al., 2022). However, the role of PRC1 subunits including CBX4, in EV71 replication and pathogenesis remains unclear. In present study, our initial results revealed that overexpression of CBX4 promoted the expression of EV71 viral proteins, VP1 mRNA, and viral replication intermediate dsRNA, consequently increasing the viral titers in the cell supernatants. Similarly, knockdown of CBX4 inhibited the replication of EV71. These findings suggest that CBX4 is necessary for EV71 replication and maintenance of infection in cells. Previous researches reported that CBX4 SUMOylates target proteins via two SIMs or regulates chromatin homeostasis through N-terminal chromodomain, respectively (Li et al., 2014; Luis et al., 2011). Here, we revealed that CBX4 specifically relies on its SUMOylation E3 ligase function rather than PRC1-related function to regulate EV71 replication.

Posttranslational modifications (PTMs) of viral proteins act crucial roles in viral replication and pathogenesis. For EV71, NEDD8-mediated Neddylation of VP2 attenuates VP2 stability and EV71 replication (Wang H. et al., 2022). Ubiquitin E3 ligase SPOP induces the ubiquitination and degradation of 2A, thus inhibiting EV71 infection (Zang et al., 2023). SUMOylation of viral proteins has been shown to exert important effects in viral infection and pathogenesis (Imbert and Langford, 2021; Fan et al., 2022). For example, the SUMOylation of influenza A virus (IAV) nonstructural protein 1 (NS1), nucleoprotein (NP), polymerase basic protein 1 (PB1) and matrix protein (M1) promotes the proliferation of IAV (Santos et al., 2013; Han et al., 2014; Li et al., 2021; Gao et al., 2015), while PIAS1-mediated SUMOylation of IAV basic protein 2 (PB2) suppresses viral transcription and replication (Wang G. et al., 2022). Notably, EV71 nonstructural proteins have been reported to be SUMOylation. The SUMOylation of EV71 3C enhances its degradation and diminishes the replication of EV71 (Chen et al., 2011). Another study showed that 3D can be modified by SUMO1, and with the improvement of 3D SUMO1 level, the K63-linked ubiquitination and protein stability of 3D as well as the replication of EV71 increase evidently (Liu et al., 2016). Nevertheless, the SUMO E3 ligase that mediates 3D SUMOylation is still unknown. Our results indicated that CBX4, as a SUMO E3 ligase, could interact with 3D in cytoplasm and promote the SUMO1-induced SUMOylation and K63-linked ubiquitination modifications of 3D. Besides, we also detected that CBX4 prolonged the half-life of 3D. This suggests that CBX4 may enhance the stability of 3D through the regulation of its SUMOylation and ubiquitination.

The process of SUMO modification is similar with ubiquitination, which is realized through the cascade reaction of E1 activating enzyme, E2 conjugating enzyme and E3 ligase (Imbert and Langford, 2021). Although impairment the SUMOylation of 3C potentiates EV71 replication and cell apoptosis (Chen et al., 2011), reducing SUMOylation of cells with an inhibitor of SUMO E2 conjugating enzyme, 2-D08, significantly depressed the expression of viral 3D, VP1 and 3C proteins and VP1 mRNA in EV71-infected cells, as well as the level of productive viral release to supernatants. This may be explained from the fact that 3D exhibits a more critical effect in the EV71 replication process (Jiang et al., 2011). Moreover, the antiviral effect of 2-D08 extended to the other two enteroviruses, CVB3 and PV1. In addition, 2-D08 also significantly inhibited the expression of exogenously transfected 3D and SUMOylation of 3D, hinting that 2-D08 can be as a potential antiviral drug against enterovirus infection.

However, there were some shortcomings and drawbacks in this study. Firstly, whether 3D is a direct substrate of CBX4 has not been fully investigated *in vitro*. Secondly, the function of CBX4 on the activity of 3D polymerase and its ability to bind to viral RNA was not explored. Thirdly, the modification site of CBX4 on 3D SUMOylation and the necessity of this modification site for the function of 3D were not explored. The resolution of these questions could shed light on the mechanism of CBX4 regulation of EV71 replication, which is worth for further study.

In summary, employing a EV71 infected cell model, the present study identified the host protein CBX4 as a novel regulator of EV71 replication. The results further revealed that CBX4 interacts with EV71 RNA polymerase 3D and mediates its SUMOylation, thereby enhancing the protein stability of 3D, which ultimately upregulates the replication ability and pathogenicity of EV71. Meanwhile, inhibition of the SUMOylation process significantly suppressed the expression of EV71 3D and the replication level of EV71. This study contributes to a deeper understanding of the mechanism by which SUMOylation regulates viral replication and provides new perspectives and clues for the development of anti-EV71 drugs in the clinical setting.

## Data Availability

The original contributions presented in the study are included in the article/Supplementary material, further inquiries can be directed to the corresponding author/s.
