# Correction: CBX4 facilitates EV71 replication by SUMOylation and stabilizing 3D polymerase

**DOI:** 10.3389/fmicb.2026.1835255

**Published:** 2026-04-13

**Authors:** Frontiers Production Office

**Affiliations:** Frontiers Media SA, Lausanne, Switzerland

**Keywords:** 3D polymerase, CBX4, Enterovirus 71, protein stabilization, SUMOylation

Supplemental material Data Sheet 1 was omitted. The file(s) have now been published.

The original version of this article has been updated.

