# Supplementary material for: CBX4 facilitates EV71 replication by SUMOylation and stabilizing 3D polymerase
Source: Front Microbiol. 2026 Mar 4;17:1775950. doi: 10.3389/fmicb.2026.1775950 (PMC12996142; doi:10.3389/fmicb.2026.1775950)
Supplement: Supplementary file 1 [file Data_Sheet_1.docx]

Supplementary Material

**Supplementary Figure 1**

**
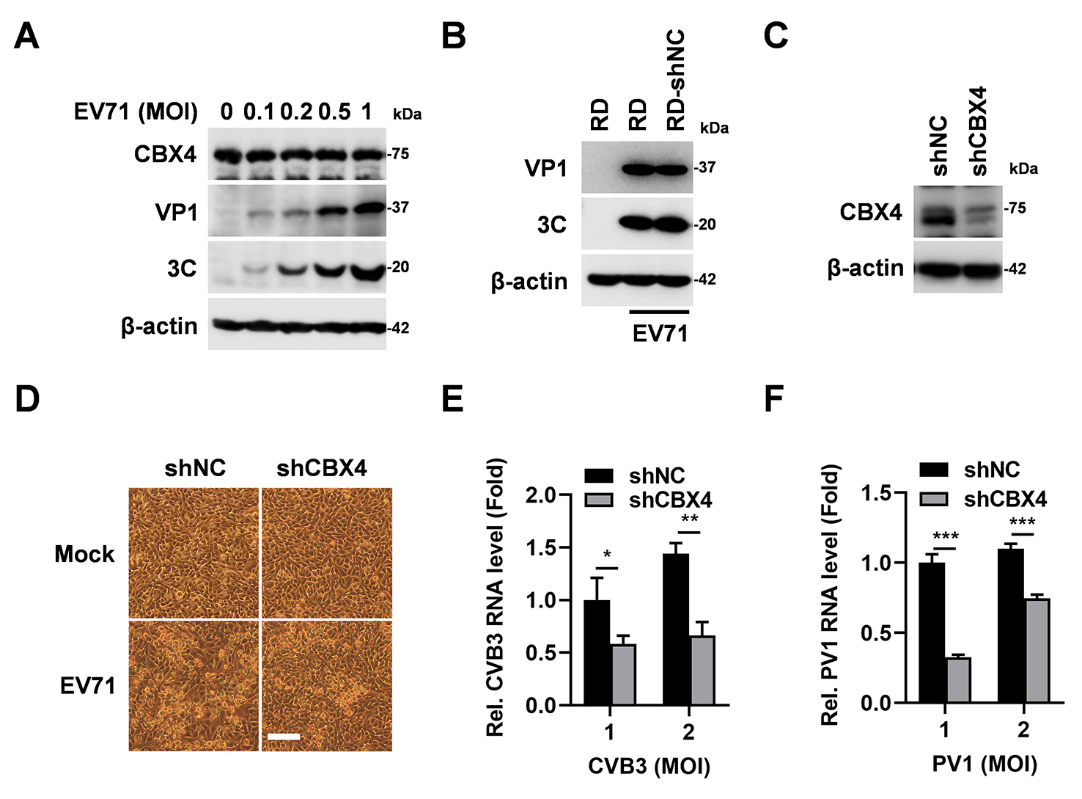
**

**Supplementary Figure 1. Knockdown of CBX4 inhibits the replication of EV71, CVB3 and PV1**

(**A**) RD cells were infected with EV71 at MOI of 0, 0.1, 0.2, 0.5 and 1 for 24 h. The expression levels of CBX4, VP1, 3C and β-actin were determined by immunoblotting analysis. (**B**) RD and RD stable expression shNC cells were infected with EV71 for 24 h, the expression levels of VP1, 3C and β-actin were determined by immunoblotting analysis. (**C**) RD cells stable expression of shNC or shCBX4 were generated, the efficiency of shRNA target to CBX4 was evaluated by Western blotting with CBX4 and β-actin antibodies. (**D**) RD stable expression shNC or shCBX4 cells were treated with EV71 (MOI = 1) for 16 h, EV71-mediated cytopathic effect was imaged under a microscope. Bar = 100 μm. (**E** and **F**) RD stably expressing shNC or shCBX4 cells were infected with CVB3 or PV1 at MOI of 1 and 2 for 24 h. The levels of CVB3 RNA (E) and PV1 RNA (F) were measured by qPCR, and the results were presented as fold induction relative to shNC cells infected with viruses at MOI of 1. The data are shown as mean ± SD. * *P*< 0.05, ** *P* < 0.01, *** *P* < 0.001.

**Supplementary Figure 2**


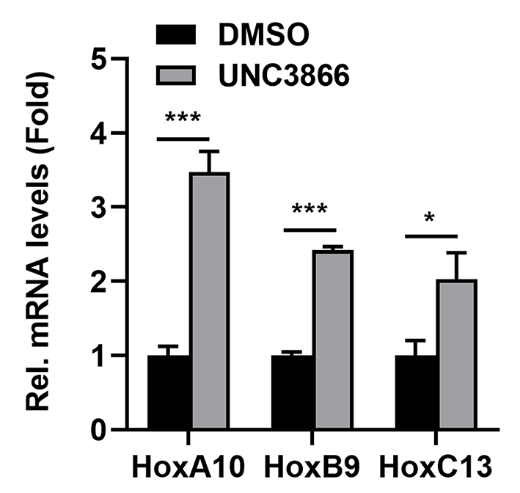


**Supplementary Figure 2. UNC3866 upregulates the expression of PRC1-inhibited target genes**

RD cells were treated with UNC3866 (500 nM) for 24 h, the mRNA levels of HoxA10, HoxB9 and HoxC13 were measured by qPCR. The results were presented as fold induction and shown as mean ± SD. * *P*< 0.05, *** *P* < 0.001.

**Supplementary Figure 3**

**
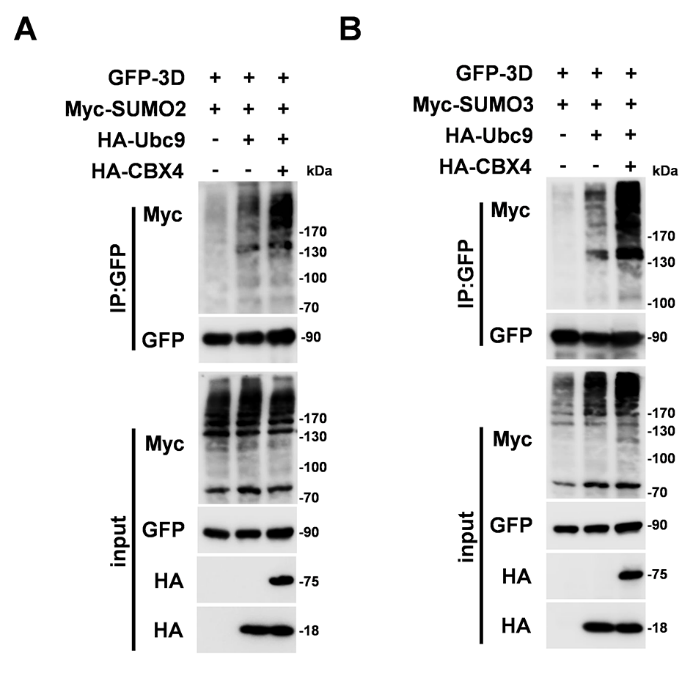
**

**Supplementary Figure 3. CBX4 facilitates the SUMOylation of 3D**

(**A** and **B**) HEK293T cells were transfected with GFP-3D, Myc-SUMO2 or Myc-SUMO3, HA-Ubc9 and HA-CBX4 plasmids as indicated, and immunoprecipitated with anti-GFP immunomagnetic beads. The IP complex was analyzed by antibodies target to Myc and GFP, and the expression levels of Myc-SUMO2 or Myc-SUMO3, GFP-3D, HA-Ubc9 and HA-CBX4 proteins in input samples were measured with Myc, GFP and HA antibodies.

**Supplementary Figure 4**


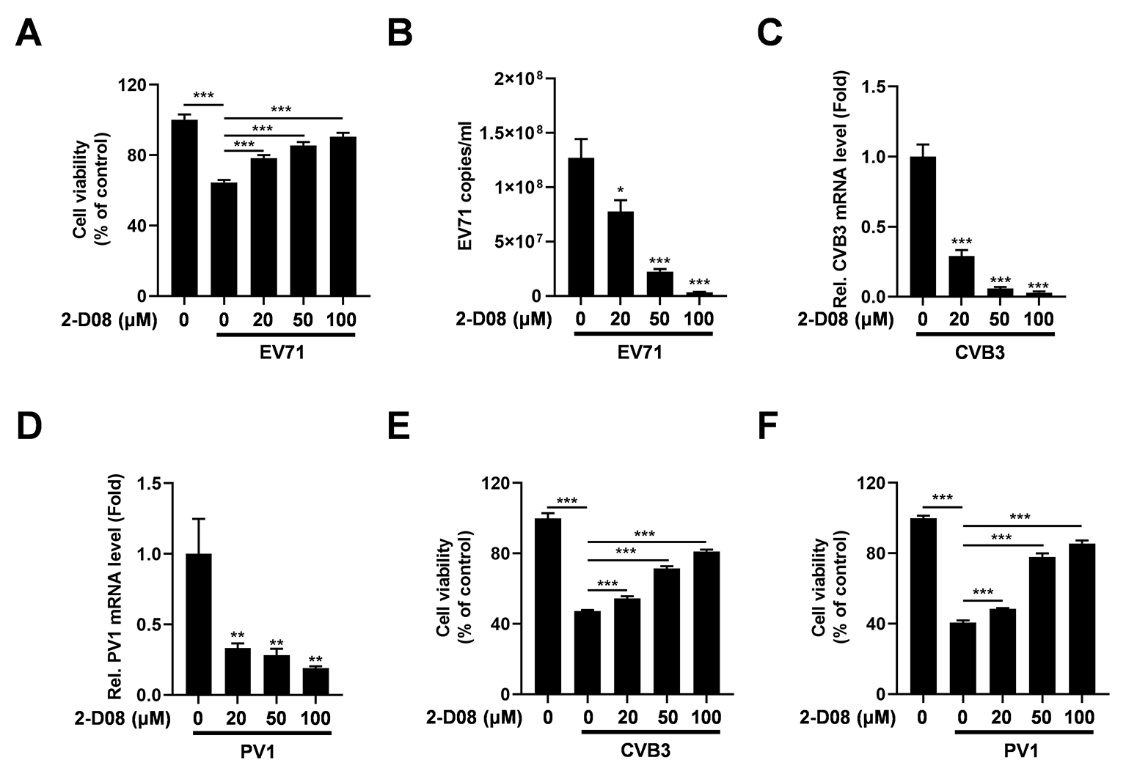


**Supplementary Figure 4. 2-D08 effectively inhibits EV71, CVB3 and PV1 infection**

(**A**) RD cells were treated with EV71 (MOI = 1) and 2-D08 (0, 20, 50, 100 μM) for 24 h. The cell viability was determined by CCK8 assay. (**B**) RD cells were treated with EV71 (MOI = 1) and 2-D08 (0, 20, 50, 100 μM) for 24 h. The culture medium supernatants were collected and the expression of EV71 copies was detected by qPCR. (**C** and **D**) RD cells were treated with CVB3 or PV1 (MOI = 1) and 2-D08 (0, 20, 50, 100 μM) for 12 h. The mRNA levels of CVB3 (C) and PV1 (D) were detected by qPCR. (**E** and **F**) RD cells were treated with CVB3 or PV1 (MOI = 1) and 2-D08 (0, 20, 50, 100 μM) for 24 h. The cell viability was determined by CCK8 assay. Data are shown as mean ± SD. * *P*< 0.05, ** *P* < 0.01, *** *P* < 0.001.
